# 3,9-Dimethyl-2,3-dihydro­spiro­[carb­az­ole-1,2′-[1,3]dithio­lan]-4(9*H*)-one

**DOI:** 10.1107/S1600536813007873

**Published:** 2013-03-28

**Authors:** Sibel Gülle, Nagihan Çaylak Delibaş, Yavuz Ergün, Tuncer Hökelek

**Affiliations:** a Celal Bayar University, Faculty of Arts and Sciences, Department of Chemistry, 45030 Muradiye, Manisa, Turkey; b Department of Physics, Sakarya University, 54187 Esentepe, Sakarya, Turkey; c Dokuz Eylül University, Faculty of Arts and Sciences, Department of Chemistry, Tınaztepe, 35160 Buca, Izmir, Turkey; d Hacettepe University, Department of Physics, 06800 Beytepe, Ankara, Turkey

## Abstract

The title compound, C_16_H_17_NOS_2_, consists of a carbazole skeleton with methyl and dithiol­ane groups as substituents. In the indole ring system, the benzene and pyrrole rings are nearly coplanar, forming a dihedral angle of 1.02 (11)°. The cyclo­hexenone ring has a twisted conformation, while the dithiol­ane ring adopts an envelope conformation with one of the CH_2_ C atoms at the flap. In the crystal, weak C—H⋯O hydrogen bonds link the mol­ecules into supra­molecular chains nearly parallel to the *c* axis. These hydrogen bonds together with weak C—H⋯π inter­actions link the molecules into a three-dimensional supramolecular network.

## Related literature

For tetra­hydro­carbazole systems present in the framework of a number of indole-type alkaloids of biological inter­est, see: Saxton (1983 ▶). For related structures, see: Hökelek *et al.* (1994 ▶, 1998 ▶, 1999 ▶, 2009 ▶); Patır *et al.* (1997 ▶); Hökelek & Patır (1999 ▶); Çaylak *et al.* (2007 ▶); Uludağ *et al.* (2009 ▶). For the isolation of carbazole alkaloids such as 3-methyl­carbazole and its several oxidized derivatives from taxonomically related higher plants, see: Chakraborty (1993 ▶); Bhattacharyya & Chakraborty (1987 ▶). For the use of 4-oxo-tetra­hydro­carbazole in the synthesis of anti­emetic drugs, central nervous system active drugs and NPY-1 antagonists, see: Littell & Allen (1973 ▶); Ping & Guoping (1997 ▶); Fabio *et al.* (2006 ▶); Kumar *et al.* (2008 ▶). For the use of 4-oxo-tetra­hydro­carbazole derivatives in the synthesis of indole alkaloids, see: Magnus *et al.* (1992 ▶); Ergün *et al.* (2000 ▶, 2002 ▶). For the synthesis of tetra­hydro­carbazolone-based anti­tumor active compounds and inhibitors of HIV integrase from 4-oxo-tetra­hydro­carbazoles, see: Li & Vince (2006 ▶). For bond-length data, see: Allen *et al.* (1987 ▶).
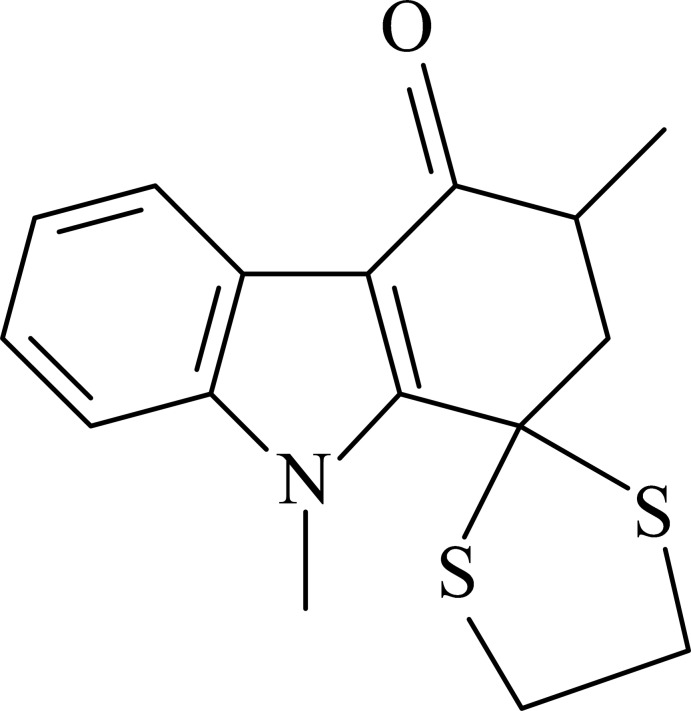


## Experimental

### 

#### Crystal data

C_16_H_17_NOS_2_
*M* *_r_* = 303.43
Orthorhombic, 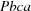

*a* = 16.8163 (3) Å
*b* = 9.8407 (2) Å
*c* = 17.2913 (4) Å
*V* = 2861.44 (10) Å^3^
*Z* = 8
Mo *K*α radiation
μ = 0.37 mm^−1^
*T* = 100 K
0.47 × 0.32 × 0.29 mm

#### Data collection

Bruker Kappa APEXII CCD area-detector diffractometer
Absorption correction: multi-scan (*SADABS*; Bruker, 2005 ▶) *T*_min_ = 0.847, *T*_max_ = 0.901
13394 measured reflections
3540 independent reflections
2912 reflections with *I* > 2σ(*I*)
*R*_int_ = 0.033

#### Refinement

*R*[*F* ^2^ > 2σ(*F* ^2^)] = 0.075
*wR*(*F* ^2^) = 0.195
*S* = 1.05
3540 reflections
183 parameters
H-atom parameters constrained
Δρ_max_ = 1.73 e Å^−3^
Δρ_min_ = −1.08 e Å^−3^

### 

Data collection: *APEX2* (Bruker, 2007 ▶); cell refinement: *SAINT* (Bruker, 2007 ▶); data reduction: *SAINT*; program(s) used to solve structure: *SHELXS97* (Sheldrick, 2008 ▶); program(s) used to refine structure: *SHELXL97* (Sheldrick, 2008 ▶); molecular graphics: *ORTEP-3 for Windows* (Farrugia, 2012 ▶); software used to prepare material for publication: *WinGX* (Farrugia, 2012) ▶ and *PLATON* (Spek, 2009) ▶.

## Supplementary Material

Crystal structure: contains datablock(s) I, global. DOI: 10.1107/S1600536813007873/xu5690sup1.cif

Structure factors: contains datablock(s) I. DOI: 10.1107/S1600536813007873/xu5690Isup2.hkl

Supplementary material file. DOI: 10.1107/S1600536813007873/xu5690Isup3.cml

Additional supplementary materials:  crystallographic information; 3D view; checkCIF report

## Figures and Tables

**Table 1:** Hydrogen-bond geometry (Å, °) *Cg*3 is the centroid of the benzene ring.

*D*—H⋯*A*	*D*—H	H⋯*A*	*D*⋯*A*	*D*—H⋯*A*
C13—H13*A*⋯O1^i^	0.99	2.60	3.483 (5)	149
C2—H2*A*⋯*Cg*3^ii^	0.99	2.89	3.813 (4)	155

Symmetry codes: (i) 
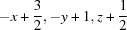
; (ii) 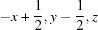.

**Fig. 1.**
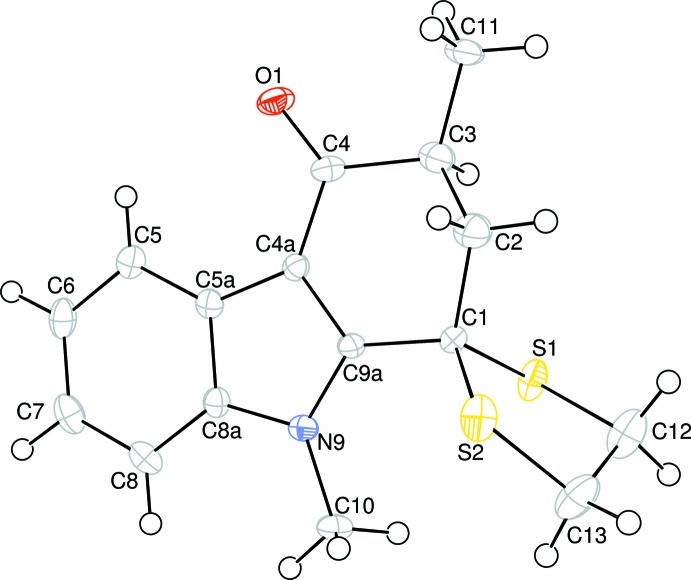
The molecular structure of the title molecule with the atom-numbering scheme. Displacement ellipsoids are drawn at the 50% probability level.

**Fig. 2.**
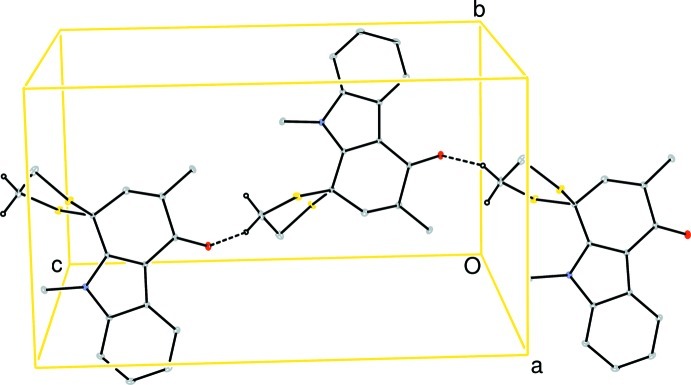
A view of the crystal packing of the title compound. The C—H···O hydrogen bonds are shown as dashed lines [H-atoms not involved in hydrogen bonding have been omitted for clarity].

## supplementary crystallographic information

### Comment

Tetrahydrocarbazole systems are present in the framework of a number of indole-type alkaloids of biological interest (Saxton, 1983). The structures of tricyclic, tetracyclic and pentacyclic ring systems with dithiolane and other substituents of the tetrahydrocarbazole core, have been reported previously (Hökelek *et al.*, 1994; Patır *et al.*, 1997; Hökelek *et al.*, 1998; Hökelek *et al.*, 1999; Hökelek & Patır, 1999). Most of the carbazole alkaloids such as 3-methylcarbazole and its several oxidized derivatives have been isolated from taxonomically related higher plants of genera *Glycosmis*, *Clausena* and *Murraya* (family *Rutaceae*) (Chakraborty, 1993; Bhattacharyya & Chakraborty, 1987). The structures of these alkaloids can vary from simple substituted carbazoles to molecules containing complex terpene moieties. Although 4-oxo-tetrahydrocarbazoles rarely occur in nature, they have been increasingly important intermediates in the syntheses of indole or carbazole alkaloids and various biologically active heterocyclic compounds because of their unique structures. For instance, 4-oxo-tetrahydrocarbazole was used in the syntheses of antiemetic drugs, central nervous system active drugs and NPY-1 antagonists (Kumar *et al.*, 2008; Fabio *et al.*, 2006; Ping & Guoping, 1997; Littell & Allen, 1973). 4-oxo-tetrahydrocarbazole derivatives have also been used in the syntheses of indole alkaloids (Magnus *et al.*, 1992; Ergün *et al.*, 2000; Ergün *et al.*, 2002). Tetrahydrocarbazolone based antitumor active compounds and inhibitors of HIV integrase were synthesized from 4-oxo-tetrahydrocarbazoles (Li & Vince, 2006). The present study was undertaken to ascertain the crystal structure of the title compound.

The molecule of the title compound, (I), (Fig. 1) consists of a carbazole skeleton with two methyl and a dithiolane groups at positions 3, N9 and 1, respectively, where the bond lengths are close to standard values (Allen *et al.*, 1987) and generally agree with those in the previously reported compounds. In all structures atom N9 is substituted.

An examination of the deviations from the least-squares planes through individual rings shows that rings B (C4a/C5a/C8a/N9/C9a) and C (C5a/C5—C8/C8a) are nearly coplanar [with a maximum deviation of -0.017 (3) Å for atom C7] with dihedral angle of B/C = 1.02 (11)°. Ring A (C1—C4/C4a/C9a) adopts twisted conformation, while the corresponding rings adopt envelope conformations in 3a,4,10,10b-tetrahydro-2H-furo[2,3-a] carbazol-5(3H)-one (Çaylak *et al.*, 2007), 3,3-ethylenedithio-3,3a, 4,5,10,10b-hexahydro-2H-furo[2,3-a]carbazole (Uludağ *et al.*, 2009) and ethyl 1-oxo-1,2,3,4-tetrahydro-9*H*-carbazole-3-carboxylate (Hökelek *et al.*, 2009). Ring A has a pseudo twofold axis running through the midpoints of C2–C3 and C4a–C9a bonds. Dithiolane ring D (S1/S2/C1/C12/C13) has a local pseudo-mirror plane running through C12 and the midpoint of the C1–S2 bond. The conformation of ring D is an envelope, with atom C12 at the flap position, 0.720 (5) Å from the mean plane through the other four atoms.

In the crystal, intermolecular weak C—H···O hydrogen bonds link the molecules into infinite chains nearly parallel to the c-axis (Table 1 and Fig. 2), in which they may be effective in the stabilization of the structure. There also exists a weak C—H···π interaction (Table 1).

### Experimental

For the preparation of the title compound, (I), a solution of 3-methyl-2,3 -dihydrospiro[carbazole-1,2'-[1,3]dithiolan]-4(9*H*)-one (1.50 g, 5.2 mmol) in dichloromethane (40 ml) was cooled to 273 K. Then, sodium hydroxide (1.5 ml, 50%), tetrabutylammonium hydrogen sulfate (0.10 g, 0.3 mmol) and methyl iodide (0.75 g, 5.3 mmol) were added. The mixture was stirred for 1 h at 273 K, the stirring was continued for 2 h at room temperature, and then washed with hydrochloric acid (50 ml, 10%). The organic layer was dried with anhydrous magnesium sulfate. The solvent was evaporated under reduced pressure and the resulting residue was recrystallized from ethyl acetate (yield; 1.50 g, 96%, m.p. 447 K).

### Refinement

The C-bound H-atoms were positioned geometrically with C—H = 0.95, 1.00, 0.99 and 0.98 Å, for aromatic, methine, methylene and methyl H-atoms, respectively, and constrained to ride on their parent atoms, with *U*_iso_(H) = k × *U*_eq_(C), where k = 1.5 for methyl H-atoms and k = 1.2 for all other H-atoms. The highest residual electron density was found 0.96 Å from C2 and the deepest hole 0.65 Å from S2.

### Crystal data

**Table:**

C_16_H_17_NOS_2_	*F*(000) = 1280
*M**_r_* = 303.43	*D*_x_ = 1.409 Mg m^−^^3^
Orthorhombic, *P**b**c**a*	Mo *K*α radiation, λ = 0.71073 Å
Hall symbol: -P 2ac 2ab	Cell parameters from 3799 reflections
*a* = 16.8163 (3) Å	θ = 2.7–28.2°
*b* = 9.8407 (2) Å	µ = 0.37 mm^−^^1^
*c* = 17.2913 (4) Å	*T* = 100 K
*V* = 2861.44 (10) Å^3^	Block, colorless
*Z* = 8	0.47 × 0.32 × 0.29 mm

### Data collection

**Table:**

Bruker Kappa APEXII CCD area-detector diffractometer	3540 independent reflections
Radiation source: fine-focus sealed tube	2912 reflections with *I* > 2σ(*I*)
Graphite monochromator	*R*_int_ = 0.033
φ and ω scans	θ_max_ = 28.5°, θ_min_ = 2.4°
Absorption correction: multi-scan (*SADABS*; Bruker, 2005)	*h* = −22→22
*T*_min_ = 0.847, *T*_max_ = 0.901	*k* = −13→10
13394 measured reflections	*l* = −23→20

### Refinement

**Table:**

Refinement on *F*^2^	Primary atom site location: structure-invariant direct methods
Least-squares matrix: full	Secondary atom site location: difference Fourier map
*R*[*F*^2^ > 2σ(*F*^2^)] = 0.075	Hydrogen site location: inferred from neighbouring sites
*wR*(*F*^2^) = 0.195	H-atom parameters constrained
*S* = 1.05	*w* = 1/[σ^2^(*F*_o_^2^) + (0.0845*P*)^2^ + 10.7607*P*] where *P* = (*F*_o_^2^ + 2*F*_c_^2^)/3
3540 reflections	(Δ/σ)_max_ < 0.001
183 parameters	Δρ_max_ = 1.73 e Å^−^^3^
0 restraints	Δρ_min_ = −1.08 e Å^−^^3^

### Special details

**Table:**

Geometry. All esds (except the esd in the dihedral angle between two l.s. planes) are estimated using the full covariance matrix. The cell esds are taken into account individually in the estimation of esds in distances, angles and torsion angles; correlations between esds in cell parameters are only used when they are defined by crystal symmetry. An approximate (isotropic) treatment of cell esds is used for estimating esds involving l.s. planes.
Refinement. Refinement of F^2^ against ALL reflections. The weighted R-factor wR and goodness of fit S are based on F^2^, conventional R-factors R are based on F, with F set to zero for negative F^2^. The threshold expression of F^2^ > 2sigma(F^2^) is used only for calculating R-factors(gt) etc. and is not relevant to the choice of reflections for refinement. R-factors based on F^2^ are statistically about twice as large as those based on F, and R- factors based on ALL data will be even larger.

### Fractional atomic coordinates and isotropic or equivalent isotropic displacement parameters (Å^2^)

**Table:**

	*x*	*y*	*z*	*U*_iso_*/*U*_eq_	
S1	0.92826 (5)	0.57718 (9)	0.92430 (5)	0.0250 (2)	
S2	0.76350 (5)	0.48874 (9)	0.95846 (6)	0.0268 (2)	
O1	0.85144 (15)	0.3722 (3)	0.64738 (14)	0.0239 (5)	
C1	0.8416 (2)	0.4921 (3)	0.88344 (19)	0.0197 (6)	
C2	0.8013 (2)	0.5688 (4)	0.8151 (2)	0.0286 (8)	
H2A	0.7473	0.5313	0.8072	0.034*	
H2B	0.7955	0.6658	0.8292	0.034*	
C3	0.8470 (3)	0.5590 (4)	0.7396 (2)	0.0289 (8)	
H3	0.9021	0.5935	0.7492	0.035*	
C4	0.85370 (19)	0.4092 (3)	0.71536 (19)	0.0191 (6)	
C4A	0.87029 (17)	0.3169 (3)	0.77841 (18)	0.0148 (6)	
C5	0.91327 (18)	0.0866 (3)	0.7158 (2)	0.0194 (6)	
H5	0.9066	0.1127	0.6633	0.023*	
C5A	0.89776 (17)	0.1783 (3)	0.77599 (19)	0.0163 (6)	
C6	0.9385 (2)	−0.0429 (3)	0.7349 (2)	0.0239 (7)	
H6	0.9489	−0.1066	0.6948	0.029*	
C7	0.9491 (2)	−0.0818 (3)	0.8121 (2)	0.0256 (7)	
H7	0.9658	−0.1718	0.8234	0.031*	
C8	0.9357 (2)	0.0079 (3)	0.8720 (2)	0.0224 (7)	
H8	0.9437	−0.0184	0.9243	0.027*	
C8A	0.90996 (18)	0.1386 (3)	0.85310 (19)	0.0170 (6)	
N9	0.89088 (16)	0.2472 (3)	0.90098 (16)	0.0181 (5)	
C9A	0.86652 (18)	0.3536 (3)	0.85548 (18)	0.0155 (6)	
C10	0.8955 (2)	0.2417 (4)	0.9851 (2)	0.0235 (7)	
H10A	0.9219	0.1574	1.0009	0.035*	
H10B	0.8418	0.2445	1.0069	0.035*	
H10C	0.9261	0.3197	1.0041	0.035*	
C11	0.8106 (2)	0.6453 (4)	0.6759 (2)	0.0255 (7)	
H11A	0.8438	0.6398	0.6294	0.038*	
H11B	0.8074	0.7400	0.6932	0.038*	
H11C	0.7571	0.6118	0.6641	0.038*	
C12	0.8746 (3)	0.6719 (5)	0.9974 (3)	0.0377 (10)	
H12A	0.9120	0.7095	1.0360	0.045*	
H12B	0.8452	0.7481	0.9734	0.045*	
C13	0.8179 (3)	0.5753 (5)	1.0353 (2)	0.0354 (9)	
H13A	0.7806	0.6254	1.0692	0.043*	
H13B	0.8474	0.5085	1.0671	0.043*	

### Atomic displacement parameters (Å^2^)

**Table:**

	*U*^11^	*U*^22^	*U*^33^	*U*^12^	*U*^13^	*U*^23^
S1	0.0233 (4)	0.0244 (4)	0.0275 (5)	−0.0063 (3)	0.0077 (3)	−0.0106 (3)
S2	0.0184 (4)	0.0257 (4)	0.0362 (5)	0.0007 (3)	0.0008 (3)	−0.0062 (4)
O1	0.0292 (12)	0.0296 (13)	0.0130 (12)	0.0025 (10)	−0.0008 (10)	0.0004 (9)
C1	0.0298 (16)	0.0154 (14)	0.0138 (15)	0.0006 (12)	−0.0039 (13)	−0.0015 (11)
C2	0.0336 (18)	0.0278 (18)	0.0243 (19)	0.0059 (15)	−0.0034 (15)	0.0004 (14)
C3	0.041 (2)	0.0244 (16)	0.0210 (18)	0.0081 (15)	−0.0011 (16)	0.0028 (14)
C4	0.0193 (14)	0.0248 (15)	0.0133 (15)	0.0020 (12)	−0.0002 (12)	0.0016 (12)
C4A	0.0144 (12)	0.0158 (13)	0.0142 (14)	−0.0020 (11)	0.0003 (11)	−0.0005 (11)
C5	0.0170 (13)	0.0209 (15)	0.0204 (16)	−0.0018 (12)	0.0045 (12)	−0.0032 (12)
C5A	0.0144 (13)	0.0169 (14)	0.0175 (15)	−0.0021 (11)	0.0031 (11)	−0.0002 (11)
C6	0.0209 (15)	0.0196 (15)	0.031 (2)	−0.0013 (12)	0.0068 (14)	−0.0083 (13)
C7	0.0216 (15)	0.0173 (14)	0.038 (2)	0.0013 (12)	0.0063 (14)	0.0014 (14)
C8	0.0207 (15)	0.0184 (15)	0.0279 (19)	0.0014 (12)	0.0019 (13)	0.0062 (13)
C8A	0.0163 (13)	0.0160 (14)	0.0187 (16)	−0.0011 (11)	0.0011 (12)	−0.0014 (12)
N9	0.0226 (12)	0.0173 (12)	0.0143 (13)	0.0013 (10)	0.0000 (11)	0.0017 (10)
C9A	0.0175 (13)	0.0155 (13)	0.0134 (15)	−0.0011 (11)	−0.0034 (11)	0.0012 (11)
C10	0.0287 (16)	0.0250 (16)	0.0167 (17)	0.0012 (14)	−0.0053 (13)	0.0050 (13)
C11	0.0318 (18)	0.0264 (16)	0.0184 (17)	0.0040 (14)	−0.0038 (14)	0.0076 (13)
C12	0.035 (2)	0.042 (2)	0.035 (2)	−0.0019 (18)	0.0037 (18)	−0.0165 (18)
C13	0.034 (2)	0.047 (2)	0.025 (2)	−0.0040 (18)	0.0074 (16)	−0.0127 (18)

### Geometric parameters (Å, º)

**Table:**

S1—C1	1.823 (3)	C6—H6	0.9500
S1—C12	1.811 (4)	C7—H7	0.9500
S2—C1	1.847 (4)	C8—C7	1.379 (5)
S2—C13	1.824 (4)	C8—H8	0.9500
O1—C4	1.231 (4)	C8A—C8	1.396 (4)
C1—C2	1.558 (5)	N9—C8A	1.390 (4)
C1—C9A	1.505 (4)	N9—C9A	1.372 (4)
C2—H2A	0.9900	N9—C10	1.458 (4)
C2—H2B	0.9900	C9A—C4A	1.382 (4)
C3—C2	1.519 (5)	C10—H10A	0.9800
C3—C11	1.520 (5)	C10—H10B	0.9800
C3—H3	1.0000	C10—H10C	0.9800
C4—C3	1.536 (5)	C11—H11A	0.9800
C4A—C4	1.446 (4)	C11—H11B	0.9800
C4A—C5A	1.441 (4)	C11—H11C	0.9800
C5—C6	1.383 (5)	C12—C13	1.498 (6)
C5—H5	0.9500	C12—H12A	0.9900
C5A—C5	1.402 (4)	C12—H12B	0.9900
C5A—C8A	1.405 (5)	C13—H13A	0.9900
C6—C7	1.400 (6)	C13—H13B	0.9900
			
C12—S1—C1	96.24 (18)	C8—C7—H7	119.3
C13—S2—C1	98.43 (17)	C7—C8—C8A	117.7 (3)
S1—C1—S2	107.72 (17)	C7—C8—H8	121.2
C2—C1—S1	114.8 (2)	C8A—C8—H8	121.2
C2—C1—S2	103.4 (2)	C8—C8A—C5A	121.6 (3)
C9A—C1—S1	108.6 (2)	N9—C8A—C5A	108.5 (3)
C9A—C1—S2	114.0 (2)	N9—C8A—C8	129.9 (3)
C9A—C1—C2	108.4 (3)	C8A—N9—C10	123.6 (3)
C1—C2—H2A	108.8	C9A—N9—C8A	108.3 (3)
C1—C2—H2B	108.8	C9A—N9—C10	128.0 (3)
C3—C2—C1	113.6 (3)	C4A—C9A—C1	124.0 (3)
C3—C2—H2A	108.8	N9—C9A—C1	126.1 (3)
C3—C2—H2B	108.8	N9—C9A—C4A	109.9 (3)
H2A—C2—H2B	107.7	N9—C10—H10A	109.5
C2—C3—C11	112.5 (3)	N9—C10—H10B	109.5
C2—C3—C4	109.4 (3)	N9—C10—H10C	109.5
C2—C3—H3	107.7	H10A—C10—H10B	109.5
C4—C3—H3	107.7	H10A—C10—H10C	109.5
C11—C3—C4	111.6 (3)	H10B—C10—H10C	109.5
C11—C3—H3	107.7	C3—C11—H11A	109.5
O1—C4—C3	122.8 (3)	C3—C11—H11B	109.5
O1—C4—C4A	122.7 (3)	C3—C11—H11C	109.5
C4A—C4—C3	114.3 (3)	H11A—C11—H11B	109.5
C5A—C4A—C4	129.4 (3)	H11A—C11—H11C	109.5
C9A—C4A—C4	123.7 (3)	H11B—C11—H11C	109.5
C9A—C4A—C5A	106.8 (3)	S1—C12—H12A	110.3
C5A—C5—H5	120.9	S1—C12—H12B	110.3
C6—C5—C5A	118.3 (3)	C13—C12—S1	107.2 (3)
C6—C5—H5	120.9	C13—C12—H12A	110.3
C5—C5A—C4A	133.7 (3)	C13—C12—H12B	110.3
C5—C5A—C8A	119.9 (3)	H12A—C12—H12B	108.5
C8A—C5A—C4A	106.4 (3)	S2—C13—H13A	110.3
C5—C6—C7	121.2 (3)	S2—C13—H13B	110.3
C5—C6—H6	119.4	C12—C13—S2	107.3 (3)
C7—C6—H6	119.4	C12—C13—H13A	110.3
C6—C7—H7	119.3	C12—C13—H13B	110.3
C8—C7—C6	121.3 (3)	H13A—C13—H13B	108.5
			
C12—S1—C1—S2	−23.5 (2)	C4—C4A—C5A—C8A	175.7 (3)
C12—S1—C1—C2	91.1 (3)	C9A—C4A—C5A—C5	−180.0 (3)
C12—S1—C1—C9A	−147.4 (3)	C9A—C4A—C5A—C8A	−0.5 (3)
C1—S1—C12—C13	45.7 (3)	C5A—C5—C6—C7	−0.5 (5)
C13—S2—C1—S1	−0.1 (2)	C4A—C5A—C5—C6	−179.0 (3)
C13—S2—C1—C2	−122.0 (3)	C8A—C5A—C5—C6	1.6 (4)
C13—S2—C1—C9A	120.4 (3)	C4A—C5A—C8A—N9	0.1 (3)
C1—S2—C13—C12	30.4 (3)	C4A—C5A—C8A—C8	179.0 (3)
S1—C1—C2—C3	75.4 (4)	C5—C5A—C8A—N9	179.6 (3)
S2—C1—C2—C3	−167.6 (3)	C5—C5A—C8A—C8	−1.5 (5)
C9A—C1—C2—C3	−46.2 (4)	C5—C6—C7—C8	−0.8 (5)
S1—C1—C9A—N9	67.1 (4)	C8A—C8—C7—C6	1.0 (5)
S1—C1—C9A—C4A	−110.8 (3)	N9—C8A—C8—C7	178.8 (3)
S2—C1—C9A—N9	−53.0 (4)	C5A—C8A—C8—C7	0.2 (5)
S2—C1—C9A—C4A	129.1 (3)	C9A—N9—C8A—C5A	0.4 (3)
C2—C1—C9A—N9	−167.6 (3)	C9A—N9—C8A—C8	−178.4 (3)
C2—C1—C9A—C4A	14.5 (4)	C10—N9—C8A—C5A	179.2 (3)
C4—C3—C2—C1	60.7 (4)	C10—N9—C8A—C8	0.4 (5)
C11—C3—C2—C1	−174.7 (3)	C8A—N9—C9A—C1	−178.9 (3)
O1—C4—C3—C2	144.3 (3)	C8A—N9—C9A—C4A	−0.7 (4)
O1—C4—C3—C11	19.1 (5)	C10—N9—C9A—C1	2.4 (5)
C4A—C4—C3—C2	−41.2 (4)	C10—N9—C9A—C4A	−179.5 (3)
C4A—C4—C3—C11	−166.4 (3)	N9—C9A—C4A—C4	−175.7 (3)
C5A—C4A—C4—O1	10.0 (5)	N9—C9A—C4A—C5A	0.8 (3)
C5A—C4A—C4—C3	−164.4 (3)	C1—C9A—C4A—C4	2.5 (5)
C9A—C4A—C4—O1	−174.3 (3)	C1—C9A—C4A—C5A	179.0 (3)
C9A—C4A—C4—C3	11.2 (5)	S1—C12—C13—S2	−50.0 (4)
C4—C4A—C5A—C5	−3.8 (6)		

### Hydrogen-bond geometry (Å, º)

Cg3 is the centroid of the benzene ring.

**Table:**

*D*—H···*A*	*D*—H	H···*A*	*D*···*A*	*D*—H···*A*
C13—H13*A*···O1^i^	0.99	2.60	3.483 (5)	149
C2—H2*A*···*Cg*3^ii^	0.99	2.89	3.813 (4)	155

Symmetry codes: (i) −*x*+3/2, −*y*+1, *z*+1/2; (ii) −*x*+1/2, *y*−1/2, *z*.
